# Temperature difference between jugular bulb and pulmonary artery is associated with neurological outcome in patients with severe traumatic brain injury: A post hoc analysis of a brain hypothermia study

**DOI:** 10.1371/journal.pone.0285525

**Published:** 2023-05-08

**Authors:** Motoki Fujita, Yasutaka Oda, Kotaro Kaneda, Tadashi Kaneko, Eiichi Suehiro, Kenji Dohi, Yasuhiro Kuroda, Hitoshi Kobata, Ryosuke Tsuruta, Tsuyoshi Maekawa

**Affiliations:** 1 Acute and General Medicine, Yamaguchi University Graduate School of Medicine, Ube, Japan; 2 Advanced Medical Emergency and Critical Care Center, Yamaguchi University Hospital, Ube, Japan; 3 Department of Emergency and Disaster Medicine, Mie University Graduate School of Medicine, Tsu, Japan; 4 Department of Neurosurgery, International University of Health and Welfare School of Medicine, Narita, Japan; 5 Department of Emergency and Critical Care Medicine, Showa University Hospital, Tokyo, Japan; 6 Department of Emergency, Disaster, and Critical Care Medicine, Kagawa University Hospital, Kagawa, Japan; 7 Department of Emergency and Critical Care Medicine/Neurosurgery, Osaka Medical and Pharmaceutical University, Takatsuki, Japan; 8 Katakura Hospital, Ube, Japan; UHC, CROATIA

## Abstract

**Background:**

The purpose of this study was to examine whether the temperature difference between the jugular bulb and pulmonary artery (ΔT_jb-pa_) is associated with the neurological outcome of patients with severe traumatic brain injury (TBI).

**Methods:**

We conducted a post hoc analysis of a multicenter randomized controlled trial of mild therapeutic hypothermia (TH, 32.0–34.0°C) or fever control (FC, 35.5–37.0°C) for the patients with severe TBI. ΔT_jb-pa_ averaged every 12 h and the variation in ΔT_jb-pa_ were compared between patients with favorable (n = 39) and unfavorable (n = 37) neurological outcomes. These values were also compared in the TH and FC subgroups.

**Results:**

The average ΔT_jb-pa_ values in patients with favorable and unfavorable outcomes were 0.24 ± 0.23 and 0.06 ± 0.36°C, respectively (P < 0.001). ΔT_jb-pa_ trended significantly higher in the favorable outcome patients than in the unfavorable outcome patients throughout the 120 h after onset of severe TBI (P < 0.001). The variation in ΔT_jb-pa_ from 0 to 72 h was significantly lower in the favorable outcome patients than in the unfavorable outcome patients (0.8 ± 0.8 vs 1.8 ± 2.5°C, respectively, P = 0.013). From 72 to 120 h, there was no significant difference in the variation in ΔT_jb-pa_. Significant differences between patients with favorable and unfavorable outcomes in ΔT_jb-pa_ and the variation in ΔT_jb-pa_ were similar in the TH subgroup, but not evident in the FC subgroup.

**Conclusions:**

A reduction in ΔT_jb-pa_ and greater variation in ΔT_jb-pa_ were associated with an unfavorable outcome in patients with severe TBI, especially those treated with TH. When treating severe TBI patients, it is important to understand that there will be differences in temperature reflecting the brain environment and the systemic temperature, depending on the severity and outcome of TBI during TH.

## Introduction

The heterogeneity of brain insult and systemic instability make understanding the pathophysiology of severe traumatic brain injury (TBI) extremely difficult. Using our data from the Brain Hypothermia Study (B-HYPO Study) in Japan [1], we recently identified several factors associated with the neurological outcomes of patients with severe TBI during the early phase of targeted temperature management (TTM), including blood glucose [2], plasma potassium [3], the partial pressure of arterial oxygen [4], a difference between mixed and jugular venous oxygen saturations [5], and a mild reduction in heart rate after tachycardia [6].

Monitoring the core body temperature as measured in the brain, jugular bulb, pulmonary artery, tympanic membrane, bladder, or rectum, is essential in patients with severe TBI. Brain temperature depends on three main factors: regional brain heat production, cerebral blood flow, and the temperature of arterial blood in the brain [7]. It has been reported that brain temperature is higher than that measured at other sites in patients with TBI because the brain is a hyper-metabolic organ [7–10]. However, monitoring brain temperature is difficult in clinical situations because it is an invasive procedure. However, the temperature in the jugular bulb reflects the metabolic status of the brain because 99% of the jugular venous blood is drained from the intracerebral vasculature [11, 12]. Mielck et al. reported that the blood temperature was significantly higher in the jugular bulb than in the aorta in patients undergoing elective coronary artery bypass graft surgery [13]. However, there are no reports of the core temperature measured in both the jugular bulb (T_jb_) and pulmonary artery (T_pa_) during TTM in patients with severe TBI.

We hypothesized that these core temperatures and the difference between them (ΔT_jb-pa_) may reflect the cerebral pathophysiology of patients with severe TBI and may be associated with their neurological outcomes. Therefore, we calculated this difference (ΔT_jb-pa_) and the variation in ΔT_jb-pa_ using a *post hoc* analysis of the B-HYPO Study in Japan [1].

## Methods

### Patients

The association between ΔT_jb-pa_ and the neurological outcomes of patients with severe TBI was examined using data from the B-HYPO Study, a prospective multicenter randomized controlled trial performed from December 2002 to September 2008 in Japan [1]. The protocol was approved by the Institutional Review Board of Yamaguchi University Hospital, as the corresponding institution. All participating hospital approved the protocol. The trial was registered on the University Hospital Medical Information Network database (UMIN-CTR, no. C000000231) in Japan and on the National Institutes of Health database (ClinicalTrials.Gov, Identifier NCT00134472) in the United States. The written informed consents were obtained from all inclusion cases. If patient was minor or unconsciousness, informed consents were obtained from a family member or guardians. Consent was waived unless a family member could consent within 2 h of the time of enrolment. In such cases, consent was obtained after the family’s arrival.

Briefly, the inclusion criteria were an age of 15–69 years and a Glasgow Coma Scale (GCS) score of 4–8. Overall, 148 patients were randomly assigned in a 2:1 ratio to either the mild therapeutic hypothermia (TH, 32.0–34.0°C, final n = 98) group or the fever control (FC, 35.5–37.0°C, n = 50) group. Neurological outcome was assessed using the Glasgow Outcome Scale (GOS) score at 6 months. The present *post hoc* analysis was performed using data from 76 of the 148 patients for whom measurements of the two core body temperatures (jugular bulb and pulmonary artery) were available. The two core body temperatures were monitored using an internal jugular venous catheter (5.5 Fr Opticath, Dainabot, Tokyo, Japan) and a pulmonary artery catheter (8Fr S-G Thermodilution Catheter, Edwards Lifescience, Tokyo, Japan), respectively, which were equipped with a thermistor. The position of the tip of the jugular venous catheter was confirmed using anterior–posterior radiography. Forty-five and thirty-one patients were treated with TH and FC, respectively.

### Treatments and monitoring of hemodynamic parameters

The treatments were performed as described in our previous article [1]. In brief, cooling was initiated within 2 h of the onset of TBI. Cooling blankets, a rapid cold fluid intravenous infusion (up to 1000 ml), and/or cold gastric lavage were used during the induction phase in both groups. Temperature control was based on T_jb_. The goal of temperature management in both groups was to achieve the target temperature within 6 h of the onset of TBI and to maintain this temperature for at least 72 h. The patients were rewarmed at a rate of <1°C/day. After rewarming, the core body temperature was maintained at <38°C until day 7 after the onset of TBI.

Sedation/analgesia was achieved with either midazolam (0.2 mg/kg/h) and a non-narcotic analgesic or with neuroleptanalgesia (droperidol 25 μg/kg/h and fentanyl 1.0 μg/kg/h). Vecuronium (0.05 mg/kg/h) or pancuronium (0.05 mg/kg/h) was also used during the body temperature control phases, as required.

Each patient’s hemodynamic status and intracranial pressure (ICP) were monitored, with the aim of maintenance at the following levels: mean arterial pressure (MAP) > 80 mmHg, ICP < 20 mmHg, cerebral perfusion pressure (CPP) > 60 mmHg, cardiac index (CI) > 2.5 L/min/m^2^, mixed venous oxygen saturation (SvO_2_) 70%–80%, and internal jugular venous oxygen saturation (SjO_2_) > 60%. The partial pressures of arterial oxygen (PaO_2_) and carbon dioxide (PaCO_2_) were maintained at >100 mmHg and 30–40 mmHg, respectively. If ICP was >20 mmHg, any treatment recommended by the Japanese guidelines was applied, including short-term hyperventilation (>30 mmHg), decompressive craniectomy, mannitol/glycerol, and/or a bolus infusion of barbiturates [14].

### Data collection and study outcome

The hemodynamic data were recorded on day 0 (before the induction of TH or FC), day 1 and day 3 after the induction of TH or FC, and 1 day after rewarming (defined as the day on which the core body temperature reached 36°C). Representative values for each day were recorded by the attending physician. Continuous data on T_jb_ and T_pa_ were recorded every 30 min throughout the measurement period. The primary outcome was the GOS score at 6 months after TBI. A favorable neurological outcome was defined as good recovery and moderate disability, and an unfavorable neurological outcome was defined as severe disability, persistent vegetative state, or death.

### Statistical analyses

In the present *post hoc* analysis, we compared the baseline characteristics, hemodynamic parameters, T_jb_, T_pa_, and ΔT_jb-pa_ between patients with favorable and unfavorable neurological outcomes. T_jb_, T_pa_, and ΔT_jb-pa_ were averaged and presented every 12 h. Data missing from these temperature records (8.02%) were replaced with the mean value of the adjacent data. The variation in ΔT_jb-pa_, or the absolute range between the maximum and minimum recorded ΔT_jb-pa_ from 0 to 72 h (induction and maintenance period of TTM) and from 72 to 120 h (after the rewarming period of TTM), was compared between patients with favorable and unfavorable neurological outcomes.

The variables are shown as means ± standard deviations (SD) or as numbers (percentages). Continuous variables at a single point were compared using Student’s *t* test and categorical variables were compared using an χ^2^ test. Continuous variables measured at multiple points were analyzed using two-way analysis of variance. When the difference was significant, the Bonferroni *post hoc* test was used to determine the specific group difference. Missing values were excluded from all analyses, except for temperature data. A P value of <0.05 was considered statistically significant. All analyses were performed using IBM SPSS Statistics for Windows version 22 (IBM SPSS Inc., Chicago, IL, USA).

## Results

### Patients’ characteristics

In this *post hoc* study, data from 45 TH and 31 FC patients were analysed. Table 1 shows the patients’ characteristics according to their neurological outcomes. The patients with favorable outcomes were significantly younger and had significantly higher GCS scores on admission than patients with unfavorable outcomes. The scores on the head CT scans did not differ significantly between the two groups. In the TH subgroup, there was no characteristic significant difference between patients with favorable and unfavorable outcomes. In the FC subgroup, the patients with unfavorable outcomes were significantly older and more were male than among the patients with favorable outcomes. There were infectious complications during TTM in five patients: one with meningitis, two with pneumonia, and two with bacteremia. There was no significant difference in infectious complications between patients with favorable and unfavorable outcomes, and the findings were similar for the FC and TH subgroups.

**Table 1:** Patient characteristics according to neurological outcomes.

	Total			TH group			FC group		
Variable	Favorable outcome	Unfavorable outcome	P value	Favorable outcome	Unfavorable outcome	P value	Favorable outcome	Unfavorable outcome	P value
	(n = 39)	(n = 37)		(n = 21)	(n = 24)		(n = 18)	(n = 13)	
Age (years)	33 ± 15	44 ± 18	0.004	34 ± 16	44 ± 18	0.059	31 ± 14	45 ± 19	0.028
Male, n (%)	31 (80)	22 (60)	0.057	14 (67)	16 (67)	1.000	17 (94)	6 (46)	0.002
Systolic blood pressure (mmHg)	142 ± 39	145 ± 31	0.742	140 ± 40	146 ± 30	0.567	145 ± 40	144 ± 35	0.899
Diastolic blood pressure (mmHg)	81 ± 22	80 ± 20	0.928	81 ± 24	81 ± 18	0.905	80 ± 21	80 ± 23	0.966
Heart rate (beats/min)	91 ± 28	96 ± 21	0.424	91 ± 29	101 ± 19	0.183	91 ± 26	85 ± 22	0.538
Glasgow Coma Scale	6.1 ± 1.3	5.5 ± 1.3	0.048	5.9 ± 1.4	5.5 ± 1.4	0.450	6.3 ± 1.2	5.3 ± 1.3	0.034
Unreactive pupil or pupils, n (%)	16 (41)	19 (51)	0.367	10 (48)	11 (46)	0.905	6 (33)	8 (62)	0.119
Surgical operation for TBI, n (%)	16 (41)	17 (46)	0.665	11 (52)	9 (38)	0.316	5 (28)	8 (62)	0.060
Injury severity score	25 ± 8	25 ± 8	0.979	26 ± 9	26 ± 9	0.922	25 ± 8	25 ± 6	0.953
AIS score for head	4.1 ± 0.6	4.4 ± 0.7	0.097	4.1 ± 0.7	4.4 ± 0.6	0.239	4.1 ± 0.6	4.4 ± 0.8	0.268
Scores on head CT scans			0.514			0.489			0.269
Diffuse injury grade I, n (%)	2 (5)	0 (0)		1 (5)	0 (0)		1 (6)	0 (0)	
Diffuse injury grade II, n (%)	14 (36)	10 (27)		6 (29)	7 (29)		8 (44)	3 (23)	
Diffuse injury grade III, n (%)	6 (15)	8 (22)		2 (9)	6 (25)		4 (22)	2(15)	
Diffuse injury grade IV, n (%)	0 (0)	0 (0)		0 (0)	0 (0)		0 (0)	0 (0)	
Evacuated mass, n (%)	16 (41)	17 (46)		11 (52)	9 (38)		5 (28)	8 (62)	
Complications of infection	1 (2.6)	4 (10.8)	0.147	1 (4.8)	3 (12.5)	0.363	0 (0)	1 (7.7)	0.232

TH; Therapeutic hypothermia, FC; Fever control, AIS; Abbreviated Injury Scale.

### Hemodynamic parameters

In this study, the median intervals from the onset of TBI and data collection on days 0, 1, and 3 of treatment and 1 day after rewarming were 6.1 (2.0–9.2) h, 25 (19–29) h, 72 (67–76) h, and 162 (121–209) h, respectively. Tables 2–4 show the hemodynamic parameters of the patients in both the TH and FC subgroups, the TH subgroup, and the FC subgroup, respectively.

**Table 2:** Comparison of hemodynamic parameters in total patients with favorable and unfavorable neurological outcome.

Variable	Day 0		Day 1		Day 3		1 Day after Rewarming	
	Favorable outcome	Unfavorable outcome	Favorable outcome	Unfavorable outcome	Favorable outcome	Unfavorable outcome	Favorable outcome	Unfavorable outcome
MAP (mmHg)	98 ± 18	85 ± 18*	92 ± 13	90 ± 16	102 ± 15	93 ± 14**	101 ± 12	96 ± 13
ICP (mmHg)	15 ± 11	16 ± 15	14 ± 6	20 ± 30	15 ± 7	18 ± 20	18 ± 7	22 ± 25
CPP (mmHg)	87 ± 19	69 ± 26**	73 ± 21	67 ± 28	83 ± 24	71 ± 31	83 ± 14	71 ± 31
SjbO_2_ (%)	57 ± 11	64 ± 22	69 ± 9	72 ± 18	71 ± 13	73 ± 16	71 ± 9	70 ± 17
CVP (mmHg)	6 ± 4	5 ± 3	8 ± 3	5 ± 8	8 ± 4	7 ± 7	6 ± 4	6 ± 4
CI (L/min/m^2^)	3.2 ± 1.3	3.7 ± 2.0	3.6 ± 1.3	3.6 ± 1.0	3.8 ± 1.4	3.7 ± 0.9	5.1 ± 2.1	4.0 ± 0.9
SvO_2_ (%)	72 ± 8	71 ± 11	78 ± 7	78 ± 9	81 ± 5	79 ± 6	79 ± 4	75 ± 12
SVRI (dynes/s/cm^–5^)	833 ± 419	670 ± 285	739 ± 354	777 ± 281	712 ± 267	673 ± 256	548 ± 223	665 ± 276

Values are means ± standard deviations. MAP, mean arterial pressure; ICP, intracranial pressure; CPP, cerebral perfusion pressure; SjbO2, jugular bulb venous oxygen saturation; CVP, central venous pressure; CI, cardiac index; SvO2, mixed venous oxygen saturation; SVRI, systemic vascular resistance index; Jb, jb, jugular bulb; PA, pa, pulmonary artery; br, brain; T, temperature; TD, temperature difference.

*P < 0.05 vs Favorable outcome,

**P < 0.01 vs Favorable outcome.

**Table 3:** Comparison of hemodynamic parameters in patients with favorable and unfavorable neurological outcome in the TH group.

Variable	Day 0		Day 1		Day 3		1 Day after Rewarming	
	Favorable outcome	Unfavorable outcome	Favorable outcome	Unfavorable outcome	Favorable outcome	Unfavorable outcome	Favorable outcome	Unfavorable outcome
MAP (mmHg)	95 ± 13	82 ± 16*	89 ± 12	89 ± 18	100 ± 14	90 ± 13*	101 ± 10	96 ± 12
ICP (mmHg)	12 ± 9	19 ± 18	12 ± 4	23 ± 38	14 ± 6	19 ± 25	16 ± 5	20 ± 22
CPP (mmHg)	84 ± 18	66 ± 26*	74 ± 15	65 ± 31	86 ± 15	69 ± 35	84 ± 13	76 ± 27
SjbO_2_ (%)	57 ± 7	64 ± 20	67 ± 11	70 ± 18	73 ± 15	71 ± 19	70 ± 9	65 ± 19
CVP (mmHg)	5 ± 3	5 ± 3	7 ± 3	6 ± 9	9 ± 4	7 ± 9	7 ± 4	5 ± 3
CI (L/min/m^2^)	2.4 ± 0.6	4.0 ± 2.3	2.7 ± 0.6	3.6 ± 0.9	3.2 ± 0.7	3.7 ± 0.9	4.8 ± 1.3	4.3 ± 0.9
SvO_2_ (%)	70 ± 8	67 ± 13	75 ± 7	78 ± 12	82 ± 4	77 ± 7	80 ± 5	72 ± 15
SVRI (dynes/s/cm^–5^)	1066 ± 501	676 ± 319	991 ± 329	752 ± 286	852 ± 249	668 ± 234	631 ± 264	674 ± 243

Values are means ± standard deviations. MAP, mean arterial pressure; ICP, intracranial pressure; CPP, cerebral perfusion pressure; SjbO2, jugular bulb venous oxygen saturation; CVP, central venous pressure; CI, cardiac index; SvO2, mixed venous oxygen saturation; SVRI, systemic vascular resistance index; Jb, jb, jugular bulb; PA, pa, pulmonary artery; br, brain; T, temperature; TD, temperature difference.

*P < 0.05 vs Favorable outcome,

**P < 0.01 vs Favorable outcome.

**Table 4:** Comparison of hemodynamic parameters in patients with favorable and unfavorable neurological outcome in the FC group.

Variable	Day 0		Day 1		Day 3		1 Day after Rewarming	
	Favorable outcome	Unfavorable outcome	Favorable outcome	Unfavorable outcome	Favorable outcome	Unfavorable outcome	Favorable outcome	Unfavorable outcome
MAP (mmHg)	100 ± 23	91 ± 21	95 ± 13	91 ± 13	104 ± 15	97 ± 15	100 ± 15	95 ± 15
ICP (mmHg)	17 ± 12	11 ± 5	15 ± 6	14 ± 9	17 ± 9	16 ± 5	21 ± 9	26 ± 30
CPP (mmHg)	90 ± 21	76 ± 25	71 ± 27	73 ± 22	79 ± 32	76 ± 20	82 ± 15	61 ± 38
SjbO_2_ (%)	56 ± 15	64 ± 27	71 ± 7	76 ± 19	70 ± 10	77 ± 12	71 ± 11	77 ± 13
CVP (mmHg)	8 ± 4	5 ± 3	8 ± 3	3 ± 2	7 ± 4	6 ± 4	6 ± 4	9 ± 6
CI (L/min/m^2^)	3.9 ± 1.3	3.2 ± 1.3	4.3 ± 1.3	3.6 ± 1.3	4.4 ± 1.6	3.6 ± 1.0	5.4 ± 2.6	3.5 ± 0.6
SvO_2_ (%)	76 ± 7	76 ± 6	81 ± 4	79 ± 6	81 ± 6	81 ± 3	78 ± 4	80 ± 8
SVRI (dynes/s/cm^–5^)	625 ± 170	660 ± 258	514 ± 188	820 ± 310	588 ± 228	682 ± 330	474 ± 160	649 ± 367

Values are means ± standard deviations. MAP, mean arterial pressure; ICP, intracranial pressure; CPP, cerebral perfusion pressure; SjbO2, jugular bulb venous oxygen saturation; CVP, central venous pressure; CI, cardiac index; SvO2, mixed venous oxygen saturation; SVRI, systemic vascular resistance index; Jb, jb, jugular bulb; PA, pa, pulmonary artery; br, brain; T, temperature; TD, temperature difference. *P < 0.05 vs Favorable outcome, **P < 0.01 vs Favorable outcome.

In all patients, MAP on day 0 and day 3 and CPP on day 0 were significantly higher in the patients with favorable outcome than in those with unfavorable outcomes (Table 2). However, there was no significance difference between the two groups in SjbO_2_, central venous pressure (CVP), CI, SvO_2_, or the systemic vascular resistance index (SVRI) on any day. In the TH subgroup, MAP on day 0 and day 3 and CPP on day 0 were significantly higher in the patients with favorable outcomes than in those with unfavorable outcomes (Table 3). In the FC subgroup, there were no significant differences in the hemodynamic parameters (Table 4). ICP tended to be lower among the total patients with favorable outcomes and in the TH subgroup patients with favorable outcomes than in those with unfavorable outcomes, but the difference was not statistically significant. CPP on day 0 was significantly higher among the total patients and the TH subgroup patients with favorable outcomes than in the corresponding patients with unfavorable outcomes.

### Difference between core body temperatures

**Fig 1:**
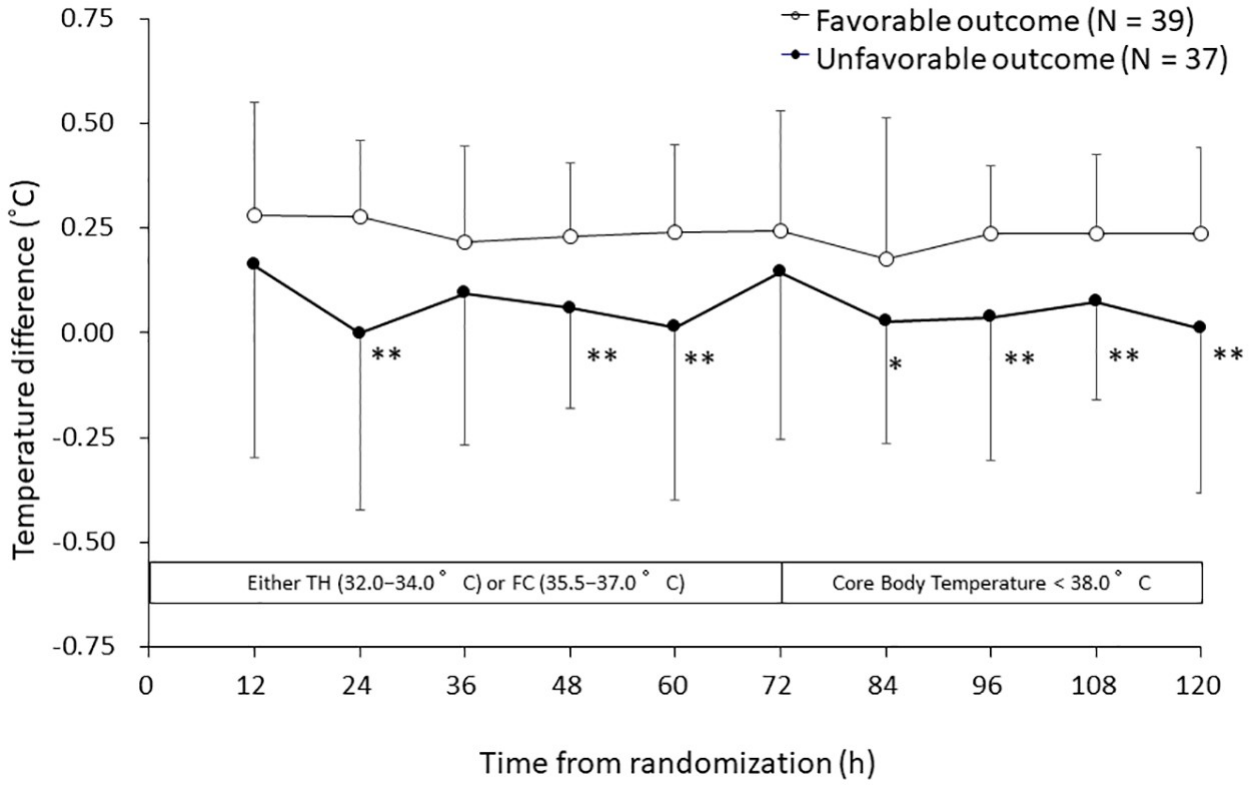
Trend in temperature difference between jugular bulb and pulmonary artery (ΔT_jb-pa_). Data shown are means ± SD. *P < 0.05, **P < 0.01.

Fig 1 shows ΔT_jb-pa_ plotted every 12 h from randomization to 120 h in all patients with favorable and unfavorable neurological outcomes. The average ΔT_jb-pa_ values in the favorable and unfavorable outcome patients were 0.24 ± 0.23 and 0.06 ± 0.36°C, respectively. ΔT_jb-pa_ was significantly larger in the favorable outcome patients than in the unfavorable outcome patients at 7 of the 10 measurement points. The significant values of ΔT_jb-pa_ were 0.28 ± 0.18°C vs 0.00 ± 0.42°C, respectively, P < 0.001 (24 h); 0.23 ± 0.18°C vs 0.06 ± 0.24°C, respectively, P = 0.001 (48 h); 0.24 ± 0.21°C vs 0.01 ± 0.41°C, respectively, P = 0.003 (60 h); 0.18 ± 0.34°C vs 0.03 ± 0.29°C, respectively, P = 0.042 (84 h); 0.24 ± 0.16°C vs 0.04 ± 0.34°C, respectively, P = 0.001 (96 h); 0.24 ± 0.19°C vs 0.07 ± 0.23°C, respectively, P = 0.002 (108 h); and 0.24 ± 0.20°C vs 0.01 ± 0.39°C, respectively, P = 0.002 (120 h).

**Fig 2:**
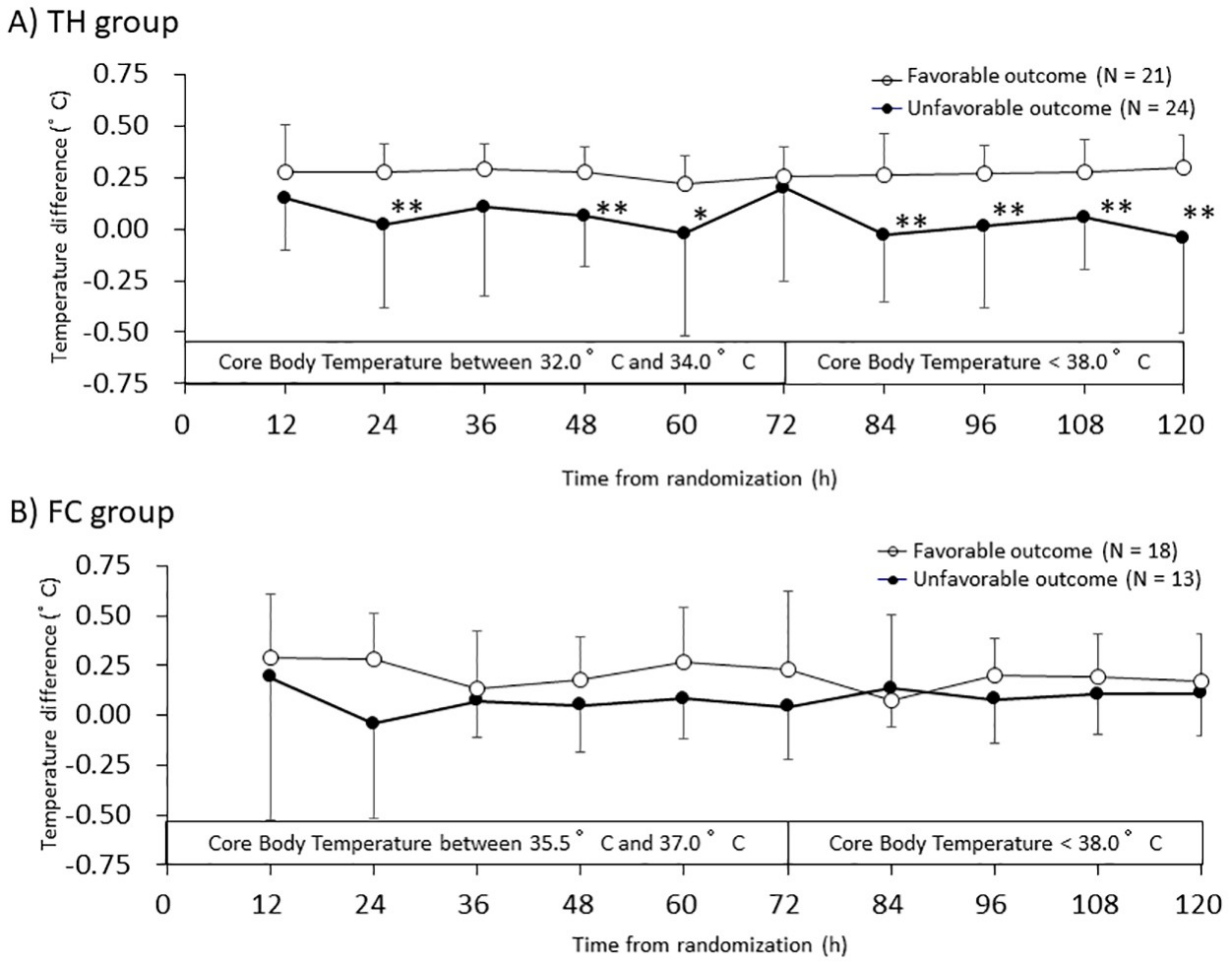
Trends in temperature difference between jugular bulb and pulmonary artery (ΔT_jb-pa_) in patients treated with therapeutic hypothermia (TH group, A) or fever control (FC group, B). Data shown are means ± SD. *P < 0.05, **P < 0.01.

Fig 2A and 2B show ΔT_jb-pa_ in the TH and FC subgroups, respectively. In the TH subgroup, ΔT_jb-pa_ was significantly larger in the favorable outcome patients than in the unfavorable outcome patients at 7 of the 10 measurement points (Fig 2A). The significant values of ΔT_jb-pa_ were 0.27 ± 0.23°C vs 0.02 ± 0.40°C, respectively, P = 0.008 (24 h); 0.28 ± 0.12°C vs 0.06 ± 0.25°C, respectively, P = 0.001 (48 h); 0.22 ± 0.14°C vs −0.02 ± 0.49°C, respectively, P = 0.036 (60 h); 0.26 ± 0.20°C vs −0.03 ± 0.32°C, respectively, P = 0.001 (84 h); 0.27 ± 0.13°C vs 0.01 ± 0.39°C, respectively, P = 0.007 (96 h); 0.27 ± 0.16°C vs 0.06 ± 0.25°C, respectively, P = 0.002 (108 h); and 0.29 ± 0.16°C vs −0.04 ± 0.46°C, respectively, P = 0.003 (120 h). In the FC subgroup, there was no significant difference at all in the trend of ΔT_jb-pa_ between the patients with favorable and unfavorable outcomes (P = 0.080; Fig 2B).

The variations in ΔT_jb-pa_ are shown in Table 5. ΔT_jb-pa_ was significantly smaller in the favorable outcome patients than in the unfavorable outcome patients in all patients (0.8 ± 0.8°C vs 1.8 ± 2.5°C, respectively, P = 0.013) and in the TH subgroup (0.6 ± 0.5°C vs 2.0 ± 2.6°C, respectively, P = 0.021). There was no significant difference in the variation in ΔT_jb-pa_ from 72 to 120 h between the patients with favorable and unfavorable outcomes in all patients, the TH subgroup, or the FC subgroup.

**Table 5:** Variation of ΔT_jb-pa_ according to neurological outcomes.

	Total			TH group			FC group		
Variable	Favorable outcome	Unfavorable outcome	P value	Favorable outcome	Unfavorable outcome	P value	Favorable outcome	Unfavorable outcome	P value
	(n = 39)	(n = 37)		(n = 21)	(n = 24)		(n = 18)	(n = 13)	
Variation of ΔT_jb-pa_ from 0 to 72 hr (°C)	0.8 ± 0.8	1.8 ± 2.5	0.013	0.6 ± 0.5	2.0 ± 2.6	0.021	0.9 ± 1.0	1.6 ± 2.1	0.236
Variation of ΔT_jb-pa_ from 72 to 120 hr (°C)	0.5 ± 0.5	0.6 ± 1.3	0.647	0.4 ± 0.3	0.7 ± 1.6	0.388	0.5 ± 0.7	0.3 ± 0.2	0.222

## Discussion

In this *post hoc* study, we have demonstrated that ΔT_jb-pa_ was significantly larger (around 0.25°C) up to 120 h in the patients with neurologically favorable outcomes than in those (close to 0°C) with unfavorable outcomes (Fig 1). Furthermore, the variation in ΔT_jb-pa_ during the induction and maintenance periods of TTM was significantly lower in the patients with neurologically favorable outcomes (Table 5). These same differences were observed in the TH subgroup but not in the FC subgroup (Fig 2A and 2B, and Table 5). This is the first report to show that ΔT_jb-pa_ reflects the neurological outcomes of patients with acute brain insult in a relatively early stage of TTM, especially during TH (Figs 1 and 2A).

In this study, ΔT_jb-pa_ was large (around 0.25°C) in the neurologically favorable outcome patients, especially during treatment with TH (Figs 1 and 2A). In humans, the cerebral metabolic rates for glucose and oxygen are much higher than those of the whole body [15], so the temperature of the venous blood in the brain and jugular bulb may be much higher than that in the other systemic venous vasculature. The difference between the brain and pulmonary arterial temperatures was reported to be 0.3 ± 0.3°C by Rossi et al. [7], and the mean ΔT_jb-pa_ was reported by Crowder et al. [11] to be 0.2°C before craniotomy in neurosurgical patients. Mielck et al. also reported that the jugular bulb (blood) temperature was much higher than the systemic core body temperature [13]. These findings are consistent with our results in all patients with favorable outcomes and those patients in the TH subgroup with favorable neurological outcomes (Figs 1 and 2A), whose values for ICP and CPP were better than those of the patients with unfavorable neurological outcomes (Tables 2 and 3).

In terms of their neurological outcomes at 6 months after the onset of severe TBI, ΔT_jb-pa_ was positive (around 0.25°C) in patients with favorable neurological outcomes but was near 0°C in those with unfavorable neurological outcomes (Figs 1, 2A and 2B). Several factors might explain this difference. First, heat clearance from the brain may have been reduced in patients with unfavorable outcomes, in whom ICP was higher and CPP was lower, although many differences were not significant (Tables 2–4). Lower CPP may induce lower CBF and lower heat clearance from the brain. Second, heat production of the injured brain region itself may have been reduced in the patients with unfavorable outcomes. In our previous sub-analysis, the difference in oxygen saturation between the mixed venous blood and the jugular bulb blood tended to decrease on days 1 and 3 in patients with unfavorable outcomes [5]. This finding indicates that the cerebral metabolic rate for oxygen (CMRO_2_) in these patients was suppressed. This phenomenon supports the present result that ΔT_jb-pa_ was significantly smaller (close to 0°C) in the neurologically unfavorable outcome patients (Figs 1 and 2A). Moreover, the thermal control center (hypothalamus) would have been damaged in the early stage of TTM by the severe TBI itself or by the regional ischemia caused by the reduction in CPP [16]. These phenomena may explain, at least in part, why ΔT_jb-pa_ approached 0°C in these patients with neurologically unfavorable outcomes (Figs 1, 2A and 2B). The results of this study did not demonstrate that ΔT_jb-pa_ is an indicator for the treatment of severe TBI, but it may at least be useful in predicting patients’ outcomes. In either case, when treating severe TBI, it should be noted that the temperature reflecting the brain environment and the systemic temperature will differ during TH depending on the severity or outcome of TBI.

In this study, the variation in ΔT_jb-pa_ during the induction and maintenance periods of TTM was significantly larger in the patients with neurologically unfavorable outcomes, especially in the TH subgroup (Table 5). The therapeutic temperature intervention itself has been reported to alter the difference (ΔT) between the brain and systemic temperatures in either direction, but prognosis has not been discussed in detail [17]. In this study, the variation in ΔT_jb-pa_ was significantly larger in patients in the TH subgroup with unfavorable outcomes. The larger variation in ΔT_jb-pa_ in patients with unfavorable outcomes may be related to the impairment of the temperature control center by the severe TBI itself and by patient care, such as a reduction in the regional cerebral blood flow. In either case, it is important to understand that any temperature control strategy that targets the systemic temperature in patients with severe TBI with unfavorable outcomes will not only alter the temperature reflecting the target organ of temperature control, the brain, but will also increase its variability.

This study had several limitations. First, the sample size was reduced at each point because data for T_jb_ and T_pa_ were not recorded for all patients. This may have biased the results of the study. Second, the physiological parameters used in the analysis were recorded only once on days 0, 1, and 3 of TTM maintenance and 1 day after rewarming, which limited the number of values recorded. Third, we did not collect blood data on infectious complications, so we could not consider the effect of infection on body temperature. However, there was no significant difference in the complication rate of infections between patients with favorable and unfavorable outcomes. Finally, we only conducted a *post hoc* analysis of data from the B-HYPO Study [1], and a prospective study is required to verify the usefulness of ΔT_jb-pa_ in evaluating the outcomes of patients with severe TBI.

## Conclusions

In this study of TTM, smaller ΔT_jb-pa_ and greater variation in ΔT_jb-pa_ were associated with unfavorable outcomes in patients with severe TBI, especially in those treated with TH. It is important to understand that the temperature reflecting the brain environment and the systemic temperature will differ, depending on the severity or outcome of TBI during TTM.

## Supporting information

S1 Fig Trends in jugular bulb temperature (T_jb_) in patients treated with therapeutic hypothermia (TH subgroup, A) or fever control (FC subgroup, B). Data shown are means ± SD. *P < 0.05.
(TIF)

S2 Fig Trends in pulmonary artery temperature (T_pa_) in patients treated with therapeutic hypothermia (TH subgroup, A) or fever control (FC subgroup, B). Data shown are means ± SD. *P < 0.05.
(TIF)

S1 Data (XLSX)
