# Supplementary figures and images for: Temperature difference between jugular bulb and pulmonary artery is associated with neurological outcome in patients with severe traumatic brain injury: A post hoc analysis of a brain hypothermia study
Source: PLoS One. 2023 May 8;18(5):e0285525. doi: 10.1371/journal.pone.0285525 (PMC10166477; doi:10.1371/journal.pone.0285525)

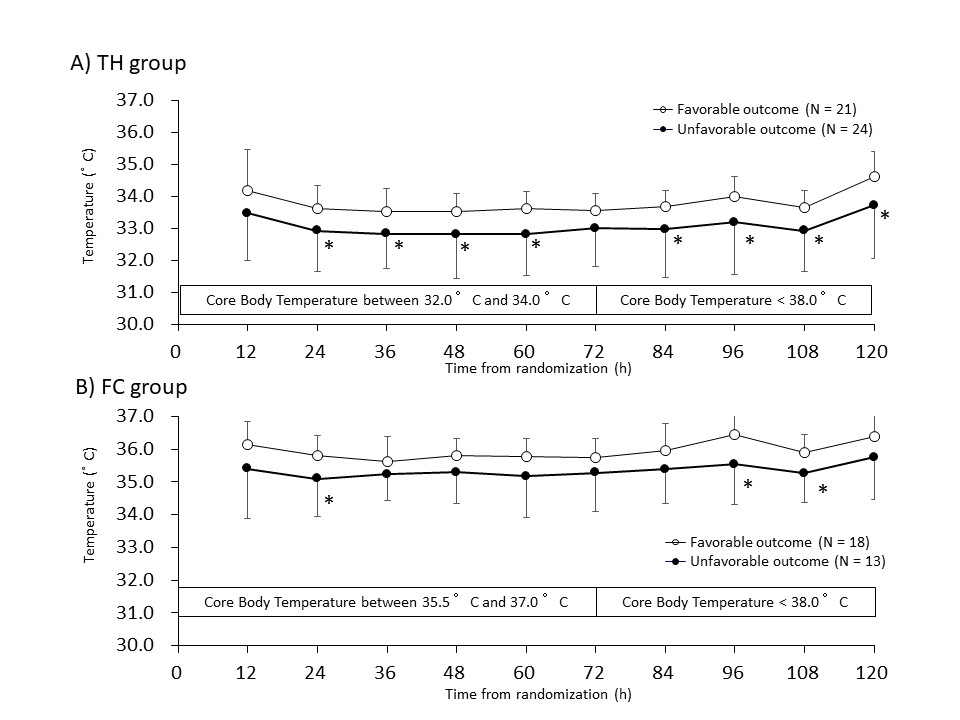

Supplement: S1 Fig — Data shown are means ± SD. *P < 0.05. (TIF) [file pone.0285525.s001.TIF]

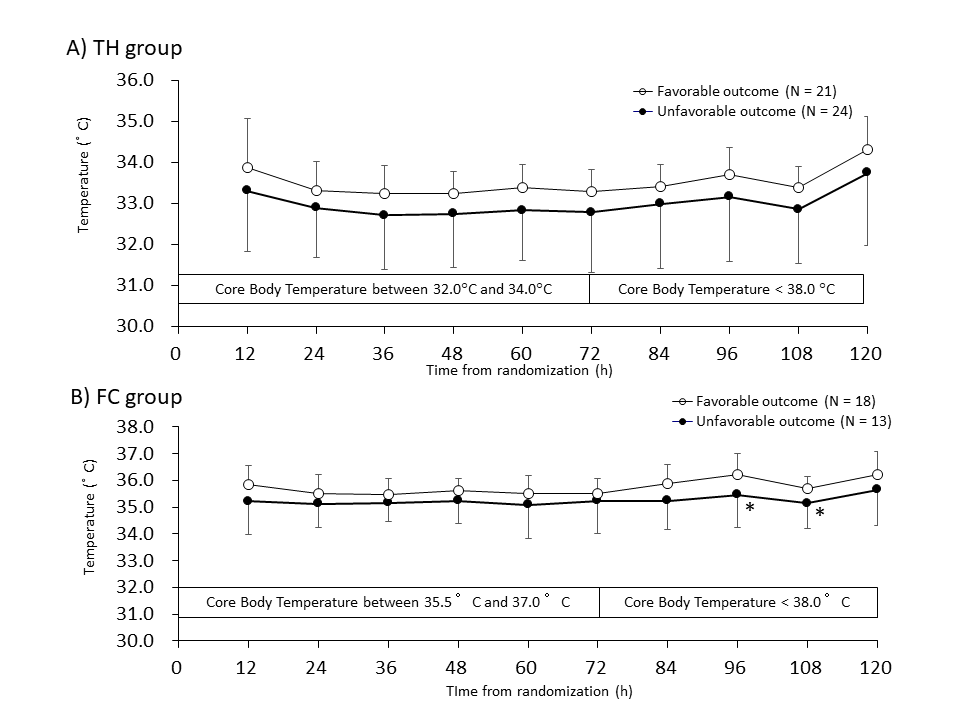

Supplement: S2 Fig — Data shown are means ± SD. *P < 0.05. (TIF) [file pone.0285525.s002.TIF]
