# Supplementary material for: Temperature difference between jugular bulb and pulmonary artery is associated with neurological outcome in patients with severe traumatic brain injury: A post hoc analysis of a brain hypothermia study
Source: PLoS One. 2023 May 8;18(5):e0285525. doi: 10.1371/journal.pone.0285525 (PMC10166477; doi:10.1371/journal.pone.0285525)
Supplement: Attachment — Submitted filename: Response to comments 20230107.docx [file pone.0285525.s004.docx]

We thank the staff at the journal office for their critical comments and useful suggestions, which have helped us to improve the manuscript considerably. As indicated in the responses below, we have considered all these comments and suggestions in revising the manuscript. In the revised manuscript, the revised text is shown in red font.

**Journal Requirements:**

**1. Please ensure that your manuscript meets PLOS ONE's style requirements, including those for file naming. The PLOS ONE style templates can be found at**

**https://journals.plos.org/plosone/s/file?id=wjVg/PLOSOne_formatting_sample_main_body.pdf and** [**https://journals.plos.org/plosone/s/file?id=ba62/PLOSOne_formatting_sample_title_authors_affiliations.pdf**](https://journals.plos.org/plosone/s/file?id=ba62/PLOSOne_formatting_sample_title_authors_affiliations.pdf)

**Response:**

We have revised the manuscript according to PLOS One’s style requirements.

**2. Please provide additional details regarding participant consent. In the ethics statement in the Methods and online submission information, please ensure that you have specified what type you obtained (for instance, written or verbal, and if verbal, how it was documented and witnessed). If your study included minors, state whether you obtained consent from parents or guardians. If the need for consent was waived by the ethics committee, please include this information.**

**Response:**

We have added the details on participant consent to the Methods section (p5, l100-109 in the revised manuscript).

**3. Please include your full ethics statement in the ‘Methods’ section of your manuscript file. In your statement, please include the full name of the IRB or ethics committee who approved or waived your study, as well as whether or not you obtained informed written or verbal consent. If consent was waived for your study, please include this information in your statement as well.**

**Response:**

We have added the full ethics statement to the Methods section (p5, l100-109 in the revised manuscript).

We are grateful to the Editor for his/her critical comments and useful suggestions, which have helped us to improve the manuscript considerably. As indicated in the responses that follow, we have taken all these comments and suggestions into account in revising the manuscript. In the revised manuscript, the revised text is shown in red font. In the responses below, we have cited the relevant page and line numbers in the revised manuscript.

**Additional Editor Comments:**

1. **Please state in which period you monitored the patients (years).**

**Response:**

The B-HYPO Study was performed between December 2002 and September 2008 in Japan. Each enrolled patient was followed-up for 6 months after enrollment. We have added the study period to the Methods section of the revised manuscript (p5, l99-100 in the revised manuscript).

2. **Why did they use neurolept sedation, which can cause deep hypotension and has a long half-life, the same as pancuronium?**

**Response:**

The patient’s core body temperature must be controlled as tightly as possible for at least 72 h, and the relatively long-acting neurolept sedation is most suitable for this. Droperidol dilates the resistance vessel, as you mentioned, and the transfusion of an adequate intravascular volume is necessary. However, we were able to maintain high cardiac output with low-dose dobutamin (a beta-1 stimulant). Fentanyl is a strong narcotic and therefore maintains intrinsic catecholamines at low levels and suppresses vascular resistance. Narcotics also prevent shivering. It is essential to maintain or increase cerebral blood flow when n managing patients with head injuries, so their cardiac output must remain stable for at least 72 h.

We prefer a relatively long-acting muscle relaxant because shivering must be prevented, especially in the early phase of therapeutic hypothermia. Thereafter, we taper off pancuronium on day 3 or a few days before the ventilator-free day, so we need not worry about the accumulation of muscle relaxant when the artificial ventilation is weaning off.

Using these methods, we can ensure a stable cardiorespiratory background for patients with severe head injuries combined with pathophysiology.

3. **You do not provide information on inflammatory parameters and possible infection as a cause of elevated temperature.**

**Response:**

We did not collect blood data on infectious complications and therefore could not consider the effects of infection on body temperature. However, the rates of infectious complication did not differ significantly between patients with favorable and unfavorable outcome. Although the patients with unfavorable outcome tended to have more infectious complications (Table 1), both the T_pa_ and T_jv_ trends were lower in patients with unfavorable outcome (S1 Fig and S2 Fig).

We have added the data on infectious complications to Table 1 (p10 in the revised manuscript) and have mentioned our inability to relate infection to body temperature in the study limitations (p16, l341-344 in the revised manuscript).

4. **The GCS in both groups is initially very low.**

**Response:**

The B-HYPO Study was a random controlled trial of patients with severe traumatic brain injury, and one of the inclusion criteria was GCS 4–8, entailing low initial GCS scores.

**5. We do not know the type of brain injury and location (hematomas, diffuse axonal injury, contusiones, tec.) because ultimately the outcome of the patient depends on it.**

**Response:**

We have added the scores on the head CT scans to Table 1 (p9 in the revised manuscript).
