# Supplementary material for: Temperature difference between jugular bulb and pulmonary artery is associated with neurological outcome in patients with severe traumatic brain injury: A post hoc analysis of a brain hypothermia study
Source: PLoS One. 2023 May 8;18(5):e0285525. doi: 10.1371/journal.pone.0285525 (PMC10166477; doi:10.1371/journal.pone.0285525)
Supplement: Peer review history

# Editor report: Decision Letter 0

**DOI:** 10.1371/journal.pone.0285525.r001

**Authors:** Nataša Kovač

21 Dec 2022

PONE-D-22-33647Temperature Difference Between Jugular Bulb and Pulmonary Artery is Associated with Neurological Outcome in Patients with Severe Traumatic Brain Injury: a Post Hoc Analysis of a Brain Hypothermia StudyPLOS ONE

Dear Dr. Fujita,

Thank you for submitting your manuscript to PLOS ONE. After careful consideration, we feel that it has merit but does not fully meet PLOS ONE’s publication criteria as it currently stands. Therefore, we invite you to submit a revised version of the manuscript that addresses the points raised during the review process.

Please submit your revised manuscript by Feb 04 2023 11:59PM. If you will need more time than this to complete your revisions, please reply to this message or contact the journal office at plosone@plos.org. When you're ready to submit your revision, log on to https://www.editorialmanager.com/pone/ and select the 'Submissions Needing Revision' folder to locate your manuscript file.

Please include the following items when submitting your revised manuscript:

- A rebuttal letter that responds to each point raised by the academic editor and reviewer(s). You should upload this letter as a separate file labeled 'Response to Reviewers'.
- A marked-up copy of your manuscript that highlights changes made to the original version. You should upload this as a separate file labeled 'Revised Manuscript with Track Changes'.
- An unmarked version of your revised paper without tracked changes. You should upload this as a separate file labeled 'Manuscript'.

If you would like to make changes to your financial disclosure, please include your updated statement in your cover letter. Guidelines for resubmitting your figure files are available below the reviewer comments at the end of this letter.

If applicable, we recommend that you deposit your laboratory protocols in protocols.io to enhance the reproducibility of your results. Protocols.io assigns your protocol its own identifier (DOI) so that it can be cited independently in the future. For instructions see: https://journals.plos.org/plosone/s/submission-guidelines#loc-laboratory-protocols. Additionally, PLOS ONE offers an option for publishing peer-reviewed Lab Protocol articles, which describe protocols hosted on protocols.io. Read more information on sharing protocols at https://plos.org/protocols?utm_medium=editorial-email&utm_source=authorletters&utm_campaign=protocols.

We look forward to receiving your revised manuscript.

Kind regards,

Nataša Kovač, MD, PhD

Academic Editor

PLOS ONE

Journal Requirements:

When submitting your revision, we need you to address these additional requirements.

1. Please ensure that your manuscript meets PLOS ONE's style requirements, including those for file naming. The PLOS ONE style templates can be found at 

https://journals.plos.org/plosone/s/file?id=wjVg/PLOSOne_formatting_sample_main_body.pdf and 

https://journals.plos.org/plosone/s/file?id=ba62/PLOSOne_formatting_sample_title_authors_affiliations.pdf

2. Please provide additional details regarding participant consent. In the ethics statement in the Methods and online submission information, please ensure that you have specified what type you obtained (for instance, written or verbal, and if verbal, how it was documented and witnessed). If your study included minors, state whether you obtained consent from parents or guardians. If the need for consent was waived by the ethics committee, please include this information.

3. Please include your full ethics statement in the ‘Methods’ section of your manuscript file. In your statement, please include the full name of the IRB or ethics committee who approved or waived your study, as well as whether or not you obtained informed written or verbal consent. If consent was waived for your study, please include this information in your statement as well. 

Additional Editor Comments:

1. Please state in which period you monitored the patients (years).

2. Why did they use neurolept sedation, which can cause deep hypotension and has a long half-life, the same as pancuronium?

3. You do not provide information on inflammatory parameters and possible infection as a cause of elevated temperature.

4. The GCS in both groups is initially very low.

5. We do not know the type of brain injury and location (hematomas, diffuse axonal injury, contusiones, tec.) because ultimately the outcome of the patient depends on it.

[Note: HTML markup is below. Please do not edit.]

[NOTE: If reviewer comments were submitted as an attachment file, they will be attached to this email and accessible via the submission site. Please log into your account, locate the manuscript record, and check for the action link "View Attachments". If this link does not appear, there are no attachment files.]

While revising your submission, please upload your figure files to the Preflight Analysis and Conversion Engine (PACE) digital diagnostic tool, https://pacev2.apexcovantage.com/. PACE helps ensure that figures meet PLOS requirements. To use PACE, you must first register as a user. Registration is free. Then, login and navigate to the UPLOAD tab, where you will find detailed instructions on how to use the tool. If you encounter any issues or have any questions when using PACE, please email PLOS at figures@plos.org. Please note that Supporting Information files do not need this step.

# Author comment: Author response to Decision Letter 0

**DOI:** 10.1371/journal.pone.0285525.r002

14 Jan 2023

We thank the staff at the journal office for their critical comments and useful suggestions, which have helped us to improve the manuscript considerably. As indicated in the responses below, we have considered all these comments and suggestions in revising the manuscript. In the revised manuscript, the revised text is shown in red font. 

Journal Requirements:

1. Please ensure that your manuscript meets PLOS ONE's style requirements, including those for file naming. The PLOS ONE style templates can be found at 

https://journals.plos.org/plosone/s/file?id=wjVg/PLOSOne_formatting_sample_main_body.pdf and https://journals.plos.org/plosone/s/file?id=ba62/PLOSOne_formatting_sample_title_authors_affiliations.pdf

Response: 

We have revised the manuscript according to PLOS One’s style requirements.

2. Please provide additional details regarding participant consent. In the ethics statement in the Methods and online submission information, please ensure that you have specified what type you obtained (for instance, written or verbal, and if verbal, how it was documented and witnessed). If your study included minors, state whether you obtained consent from parents or guardians. If the need for consent was waived by the ethics committee, please include this information.

Response: 

We have added the details on participant consent to the Methods section (p5, l100-109 in the revised manuscript).

3. Please include your full ethics statement in the ‘Methods’ section of your manuscript file. In your statement, please include the full name of the IRB or ethics committee who approved or waived your study, as well as whether or not you obtained informed written or verbal consent. If consent was waived for your study, please include this information in your statement as well. 

Response: 

We have added the full ethics statement to the Methods section (p5, l100-109 in the revised manuscript).

 

We are grateful to the Editor for his/her critical comments and useful suggestions, which have helped us to improve the manuscript considerably. As indicated in the responses that follow, we have taken all these comments and suggestions into account in revising the manuscript. In the revised manuscript, the revised text is shown in red font. In the responses below, we have cited the relevant page and line numbers in the revised manuscript.

Additional Editor Comments:

1. Please state in which period you monitored the patients (years).

Response: 

The B-HYPO Study was performed between December 2002 and September 2008 in Japan. Each enrolled patient was followed-up for 6 months after enrollment. We have added the study period to the Methods section of the revised manuscript (p5, l99-100 in the revised manuscript).

2. Why did they use neurolept sedation, which can cause deep hypotension and has a long half-life, the same as pancuronium?

Response:

The patient’s core body temperature must be controlled as tightly as possible for at least 72 h, and the relatively long-acting neurolept sedation is most suitable for this. Droperidol dilates the resistance vessel, as you mentioned, and the transfusion of an adequate intravascular volume is necessary. However, we were able to maintain high cardiac output with low-dose dobutamin (a beta-1 stimulant). Fentanyl is a strong narcotic and therefore maintains intrinsic catecholamines at low levels and suppresses vascular resistance. Narcotics also prevent shivering. It is essential to maintain or increase cerebral blood flow when n managing patients with head injuries, so their cardiac output must remain stable for at least 72 h.

We prefer a relatively long-acting muscle relaxant because shivering must be prevented, especially in the early phase of therapeutic hypothermia. Thereafter, we taper off pancuronium on day 3 or a few days before the ventilator-free day, so we need not worry about the accumulation of muscle relaxant when the artificial ventilation is weaning off. 

Using these methods, we can ensure a stable cardiorespiratory background for patients with severe head injuries combined with pathophysiology.

3. You do not provide information on inflammatory parameters and possible infection as a cause of elevated temperature.

Response:

We did not collect blood data on infectious complications and therefore could not consider the effects of infection on body temperature. However, the rates of infectious complication did not differ significantly between patients with favorable and unfavorable outcome. Although the patients with unfavorable outcome tended to have more infectious complications (Table 1), both the Tpa and Tjv trends were lower in patients with unfavorable outcome (S1 Fig and S2 Fig).

We have added the data on infectious complications to Table 1 (p10 in the revised manuscript) and have mentioned our inability to relate infection to body temperature in the study limitations (p16, l341-344 in the revised manuscript).

4. The GCS in both groups is initially very low.

Response:

The B-HYPO Study was a random controlled trial of patients with severe traumatic brain injury, and one of the inclusion criteria was GCS 4–8, entailing low initial GCS scores.

5. We do not know the type of brain injury and location (hematomas, diffuse axonal injury, contusiones, tec.) because ultimately the outcome of the patient depends on it.

Response:

We have added the scores on the head CT scans to Table 1 (p9 in the revised manuscript).

Attachment Submitted filename: Response to comments 20230107.docx Click here for additional data file.

# Peer review: Decision Letter 1

**DOI:** 10.1371/journal.pone.0285525.r003

**Authors:** Nataša Kovač

26 Apr 2023

Temperature Difference Between Jugular Bulb and Pulmonary Artery is Associated with Neurological Outcome in Patients with Severe Traumatic Brain Injury: a Post Hoc Analysis of a Brain Hypothermia Study

PONE-D-22-33647R1

Dear Dr. Motoki Fujita

We’re pleased to inform you that your manuscript has been judged scientifically suitable for publication and will be formally accepted for publication once it meets all outstanding technical requirements.

Within one week, you’ll receive an e-mail detailing the required amendments. When these have been addressed, you’ll receive a formal acceptance letter and your manuscript will be scheduled for publication.

An invoice for payment will follow shortly after the formal acceptance. To ensure an efficient process, please log into Editorial Manager at http://www.editorialmanager.com/pone/, click the 'Update My Information' link at the top of the page, and double check that your user information is up-to-date. If you have any billing related questions, please contact our Author Billing department directly at authorbilling@plos.org.

If your institution or institutions have a press office, please notify them about your upcoming paper to help maximize its impact. If they’ll be preparing press materials, please inform our press team as soon as possible -- no later than 48 hours after receiving the formal acceptance. Your manuscript will remain under strict press embargo until 2 pm Eastern Time on the date of publication. For more information, please contact onepress@plos.org.

Kind regards,

Nataša Kovač, MD, PhD

Academic Editor

PLOS ONE

Additional Editor Comments (optional):

Your work is very interesting and points to an obviously significant indicator of the pathophysiology of traumatic brain injury.

Reviewers' comments:

# Editor report: Acceptance letter

**DOI:** 10.1371/journal.pone.0285525.r004

**Authors:** Nataša Kovač

28 Apr 2023

PONE-D-22-33647R1 

Temperature difference between jugular bulb and pulmonary artery is associated with neurological outcome in patients with severe traumatic brain injury: a post hoc analysis of a brain hypothermia study 

Dear Dr. Fujita:

I'm pleased to inform you that your manuscript has been deemed suitable for publication in PLOS ONE. Congratulations! Your manuscript is now with our production department. 

If your institution or institutions have a press office, please let them know about your upcoming paper now to help maximize its impact. If they'll be preparing press materials, please inform our press team within the next 48 hours. Your manuscript will remain under strict press embargo until 2 pm Eastern Time on the date of publication. For more information please contact onepress@plos.org.

If we can help with anything else, please email us at plosone@plos.org. 

Thank you for submitting your work to PLOS ONE and supporting open access. 

Kind regards, 

PLOS ONE Editorial Office Staff

on behalf of

Dr. Nataša Kovač 

Academic Editor

PLOS ONE
